# Wall incorporation of the β-1,3-glucan cross-linking protein Pir1 in the human pathogen *Candida albicans* is facilitated by the presence of two or more Pir repeat units

**DOI:** 10.1093/femsyr/foaf042

**Published:** 2025-08-07

**Authors:** María Alvarado, Ana E Moreno-Martínez, Miguel Micó, Jesús A Gómez-Navajas, Ana Blázquez-Abellán, Verónica Mixão, Toni Gabaldón, Estibaliz Mateo, Eulogio Valentín, Piet W J De Groot

**Affiliations:** Institute for Biomedicine, ETSIAMB, University of Castilla-La Mancha, 02008 Albacete, Spain; Institute for Biomedicine, ETSIAMB, University of Castilla-La Mancha, 02008 Albacete, Spain; GMCA Research Unit, Department of Microbiology and Ecology, University of Valencia, 46100 Burjassot, Spain; Institute for Biomedicine, ETSIAMB, University of Castilla-La Mancha, 02008 Albacete, Spain; Complejo Hospitalario Universitario de Albacete (CHUA), Servicio de Salud de Castilla-La Mancha, 02008 Albacete, Spain; Barcelona Supercomputing Centre (BSC-CNS), 08034 Barcelona, Spain; Barcelona Supercomputing Centre (BSC-CNS), 08034 Barcelona, Spain; Department of Immunology, Microbiology and Parasitology, Faculty of Medicine and Nursing, University of the Basque Country (EHU), 48940 Bilbao, Spain; GMCA Research Unit, Department of Microbiology and Ecology, University of Valencia, 46100 Burjassot, Spain; Severe Infection Research Group, Medical Research Institute La Fe, 46026 Valencia, Spain; Institute for Biomedicine, ETSIAMB, University of Castilla-La Mancha, 02008 Albacete, Spain

**Keywords:** *Candida albicans*, pathogenesis, cell wall protein, Pir1, ASL protein, β-1,3-glucan

## Abstract

The Pir1 protein in the prevalent pathogenic yeast *Candidaalbicans* has been hypothesized to be important for cellular integrity by crosslinking cell wall β-1,3-glucans. However, recent studies with deletion mutants have reported contrasting results concerning its actual importance for wall integrity. Here, we present functional characterization of the two members of the Pir family (Pir1 and Pir32) as well as protein structure modeling and mutagenesis studies to elucidate how Pir1, the most important family member, is incorporated into the cell wall. Our data show that Pir1 indeed is involved in β-1,3-glucan binding but its gene deletion did not affect cellular fitness. 3D structure modeling predicts that Pir1 has a core predominantly comprised of antiparallel β-sheets, surrounded by a large loop containing a variable number of canonical Pir repeat units. Mutagenesis studies indicate that two repeat units are required and sufficient for Pir1 surface localization, wall incorporation, and Pir1-mediated glucan binding. Altogether, our work provides novel mechanistic insights into Pir1 wall incorporation and functioning, and supports its proposed role as cell wall glucan crosslinker. At the same time, *C. albicans* also may have acquired alternative means to ascertain cell wall robustness.

## Introduction

The opportunistic yeast pathogen *Candida albicans* is responsible for >50% of candidiasis cases and is the predominant cause of invasive fungal infections (Dadar et al. 2018). Systemic infections caused by *C. albicans* have a mortality rate of ∼40% and, therefore, it represents a serious public health challenge with large medical and economical importance, as highlighted by the recently published Fungal Priority Pathogens list of the WHO (World Health Organization 2022). *Candida albicans* is a diploid polymorphic yeast that is present as a commensal within the microbiome found on mucosal surfaces in the gastrointestinal, respiratory, and genitourinary tracts of most human beings (Sardi et al. 2013). *Candida albicans* can adapt to host environmental changes very quickly, even when nutrient bioavailability is restricted (Miramon and Lorenz 2017). Enhanced growth of *C. albicans* due to an imbalance in the host microbiome or immune status may result in candidiasis (Schille et al. 2025).

*Candida albicans* has a large arsenal of virulence factors that contribute to making it a successful pathogen, many of them related to the cell wall (Klis et al. 2009). The cell wall is made up of four main types of molecules. The polysaccharides β-1,3-glucan (the most abundant wall molecule), β-1,6-glucan, and chitin are present in a protective tension-bearing inner layer. This is surrounded by an outer layer mostly comprised of covalently-bound mannoproteins, which represent 30%–40% of the cell wall biomass (Kapteyn et al. 2000, Klis et al. 2001). The cell wall performs important functions in controlling *Candida* adhesion to and penetration into host tissue, stress tolerance, and morphogenesis, among others (Masuoka 2004).

Fungal covalently-bound cell wall proteins (CWPs) can be divided into two categories depending on how they are attached to the polysaccharide network. Most proteins attach to β-1,6-glucan molecules through glycosylphosphatidylinositol (GPI)-remnants and may project outward to interact with the external environment. On the other hand, a minor group of proteins, among which are Pir proteins (proteins with internal repeats), is directly bound to β-1,3-glucans in the inner skeletal layer and can be released by mild-alkali treatment (De Groot et al. 2004, De Groot et al. 2005, Klis et al. 2006). Pir proteins have been quite extensively characterized in *Saccharomyces cerevisiae* and are described as preproproteins that contain a Kex2 protease recognition site for protein maturation (Mormeneo et al. 2008, Grbavac et al. 2017), a repeated sequence (Q[IV]XDGQ[IVP]Q) that is involved in the formation of an ester linkage between the γ-carboxyl of a glutamate residue in the repeat and a hydroxyl group of β-1,3-glucan (Ecker et al. 2006), and a C-terminal domain with four conserved cysteines (Mrsă et al. 1997). To date, the ester linkage between a Pir repeat and β-1,3-glucan has only been demonstrated for *S. cerevisiae* Cis3/Pir4, a protein with two repeats (of which the second is atypical). However, this is believed to be the general mechanism of linking Pir proteins to cell wall β-1,3-glucan. Therefore, Pir proteins with multiple repeats are proposed to act as crosslinkers of β-1,3-glucan chains, thereby strengthening the cell wall matrix.

Multiple deletion of *PIR1, PIR2, PIR3*, and *PIR4*, the four most important of the five *PIR* genes in *S. cerevisiae*, showed that they are not essential for growth but are important for cell wall integrity, osmotic tolerance, and growth at low pH or in the presence of antifungal agents (Mrsă and Tanner 1999). The importance of Pir proteins for cell wall integrity is further evidenced by the fact that *PIR* genes show elevated expression under cell wall stress conditions (Smits et al. 1999, Zakrzewska et al. 2005). In addition, *S. cerevisiae* also contains five GPI proteins (Cwp1, Cwp2, Tip1, Tir1, and Ans1) that contain a single Pir repeat, suggesting that these proteins may further strengthen the cell wall by forming crosslinks between β-1,3- and β-1,6-glucan molecules (De Groot et al. 2005).

Pir proteins are not unique for *S. cerevisiae*, in fact, they seem conserved in the subphylum *Saccharomycotina*, and Pir-like proteins have also been described in some other (mycelial) species such as *Neurospora crassa* and *Magnaporthe grisea*, albeit with a different protein organization (De Groot et al. 2005). The pathogenic yeast *C. albicans* contains two Pir-like proteins, Pir1 and Pir32, of which the former is routinely being identified in wall proteomic studies (De Groot et al. 2004, Sosinska et al. 2011, Sorgo et al. 2013). *Candida albicans* reference strain SC5314 encodes two different allelic versions of *PIR1*, containing seven and nine Pir repeats, respectively (Butler et al. 2009). *PIR1* allelic versions with five or eight repeats have also been observed in clinical isolates (Kim et al. 2022). Pir32 contains only a single repeat unit, and this may explain why the protein never has been identified in the cell wall. GPI proteins with Pir repeats are absent in *C. albicans*, underpinning the proposed importance of Pir1 glucan crosslinking in attaining sufficient cell wall strength. The increased wall incorporation of Pir1 observed under various stress conditions (Sorgo et al. 2011, Ene et al. 2012, Heilmann et al. 2013) also supports this idea.

Initial studies with *PIR1* deletion mutants in *C. albicans* suggested it to be an essential gene exhibiting a severe haploinsufficiency phenotype, including abnormal agglutination, slow growth rates, and hypersensitivity to cell wall disrupting agents such as Congo red (CR) or Calcofluor white (CFW) (Martínez et al. 2004). However, more recently Kim et al. (2022) reported that simultaneous homozygous deletion of *PIR1* and *PIR32* in *C. albicans* had only marginal effects on cell physiology.

Here, we analyzed the relevance of both *C. albicans* Pir proteins for cell integrity and present a detailed study of Pir1 wall incorporation and functionality through mutagenesis studies with truncated and site-mutated versions of the protein. Our data support that, although null mutants do not show drastic growth defects, Pir1 has a function in making connections to β-1,3-glucan. We also show that two repeat units are needed and sufficient for covalent wall incorporation and functionality of Pir1. Further, we pinpoint some repeat residues that seem crucial for covalent Pir1 wall incorporation. Our data are corroborated by protein structure modeling and supports the hypothesis that the repeat units govern the incorporation of Pir proteins to enhance cell wall strength.

## Materials and methods

### Strains and culturing conditions

*Candida albicans* reference strain SC5314 (Odds et al. 2004) was used for the genetic work described in this study. Clinical isolates obtained from local hospitals in Albacete, Valencia, and Bilbao were used for studying Pir1 repeat unit variations. Strains were grown and maintained in YPD (1% yeast extract, 2% peptone, 2% glucose) at 37°C unless stated otherwise.

### Generation of deletion mutants

A deletion construct harboring up- and downstream flanks about 0.5 kb of the long allele of *PIR1* was prepared in the *C. albicans*-optimized *SAT1*-flipper plasmid pSFS2 (Reuss et al. 2004) using proofreading Kapa DNA polymerase (KAPA Biosystems). After sequence verification (STAB-Vida), the linearized cassette was transformed into *C. albicans* together with CRISPR–Cas9 RNP complex elements, as described (Grahl et al. 2017). Transformants were analyzed by PCR to select deletion mutants and to determine if the cassette had integrated into one or both *PIR1* alleles. To this end, primers located inside the deleted gene fragment up- and downstream of the repetitive sequences were used (Supplementary Table S1), allowing us also to differentiate between the two alleles of *PIR1*. Multiple selected mutants (homozygous and heterozygous) were grown in liquid yeast nitrogen base (YNB) medium (0.17% YNB without amino acids and 0.5% ammonium sulfate), supplemented with 2% maltose to induce expression of the *FLP* gene from the *MAL2* promoter to excise the inserted cassette, followed by selection and verification of flip-outs, as previously described (Reuss et al. 2004). The knockout procedure, flippase recombination, and verification (PCR and immunoblot analysis) of the *pir1*Δ mutants is exemplified in Fig. S1. In addition, whole genome sequencing (WGS) using an Illumina paired-end sequencing strategy (Mixao and Gabaldon 2020) confirmed that the only alteration in the genome of the homozygous mutant strain with respect to the wild-type reference strain in NCBI (BioProject PRJNA432884) is the absence of *PIR1*, being replaced by 34 bp corresponding to the flippase recognition target site. Deletion mutants of *PIR32* in SC5314 and in *pir1*Δ/Δ background (*pir1*Δ/Δ *pir32* Δ/Δ double mutants) were generated using the same methodology. All phenotypic assays with deletion mutants were performed with at least two independent homozygous mutants.

### Complementation and mutagenesis studies

A reintegrant strain with the short (*PIR1*_B) allele containing seven repeats was generated to restore the *PIR1* gene function in the *pir1*∆/∆ null mutant. The *PIR1* gene plus promoter and terminator regions (positions −1090 to +1549) were PCR amplified from strain SC5314 and cloned into plasmid CIpSAT2 (Moreno-Ruiz et al. 2009), a derivative of the CIp10 plasmid containing *CaSAT1* as selection marker (Murad et al. 2000). Verified, linearized constructs were transformed into the *pir1*∆/∆ mutant. The generated plasmid was also used to obtain truncated *PIR1* versions containing 0, 1, or 2 repeat units by fusion PCR. In the latter construct, specific point-mutations in both repeats were introduced by site-directed mutagenesis using QuikChange II (Agilent) following the manufacturer´s guidelines. Nourseothricin-resistant transformants containing reintegrated *PIR1* gene versions were verified by PCR using primers flanking the integrated cassette (Supplementary Table S1).

### Growth rate, cell morphology, and surface hydrophobicity

For determination of growth rates, cells from overnight (o/n) precultures were diluted to an OD_600_ = 0.1 in fresh YPD and incubated at 37°C and 200 rpm for 12 h, measuring the OD_600_ every hour. Cell morphology was analyzed at the end of the experiment by optical microscopy using a Leica DM1000 microscope mounted with a MC170 HD digital camera (Leica Biosystems).

Filamentation was tested by adding cells (5% (v/v)) from an o/n preculture and fetal bovine serum (20% (v/v); Sigma) to fresh YPD and following germ tube formation every hour during a 6-h incubation at 37°C.

Agar invasion was tested by spotting 10 µl of precultured (o/n) cells on YPD agar and incubating for 72 h at 37°C with normal air pressure or under vacuum. Agar invasion was observed each day after washing plates with mQ water. Morphology of cells inside cut agar slices was analyzed by optical microscopy.

Cell surface hydrophobicity was determined by measuring the relative distribution of yeast cells in a two-phase system, as described (Reithofer et al. 2021).

### Sensitivity assays

Minimum inhibitory concentrations (MIC) of the antifungal compounds amphotericin B (AmBisome), micafungin (Astellas Pharma), caspofungin (MSD), isavuconazole (Basilea), and fluconazole (Acros), and SDS (Merck) were determined as described (Reithofer et al. 2021) and following EUCAST guidelines (EUCAST 2023).

Sensitivity to the cell wall perturbing agents CR (Sigma; 100 μg/mL) and CFW (Sigma; 150 μg/mL), heat shock (15 min at 65°C), and NaCl (500 mM) and LiCl (200 mM) was analyzed using drop tests. Briefly, o/n precultures were diluted to an OD_600_ = 1. From these, ten-fold serial dilutions were prepared, and 4 μl aliquots were spotted on YPD plates with or without the tester compound. Plates were photographed after 24 h of incubation at 37°C.

Sensitivity to the β-1,3-glucanase-containing enzyme preparation Zymolyase-20T (Ambsio) was determined as described previously (Reithofer et al. 2021).

### Biofilm formation and adhesion

For analysis of the biofilm formation capacity, the OD_600_ of o/n precultures was adjusted to 2. Polystyrene 96-wells plates were filled with 50 μl of the cell suspensions and 150 μl of fresh Roswell Park Memorial Institute 1640 (RPMI) or YPD medium. After a 24-h incubation at 37°C in a humid environment, the biofilm mass was quantified with the crystal violet (CV) assay, as described (Reithofer et al. 2021, Alvarado et al. 2024).

Short time (4 h) adherence to polystyrene and to polysaccharides with same linkages as fungal cell wall molecules was determined as described previously (Fernández-Pereira et al. 2021, Alvarado et al. 2024). Briefly, polystyrene 12-well plates, without or after coating (by evaporation) with laminarin (β-1,3-glucan, Alfa Aesar J66193; 500 mg/ml in milli-Q water), pustulan (β-1,6-glucan, Calbiochem 540 501; 500 mg/ml in 50 mM potassium acetate buffer) or chitin (Sigma C7170; 250 mg/ml in 1% acetic acid), were incubated with 1 × 10^6^ cells from o/n precultures in phosphate-buffered saline (PBS) (or PBS alone). After a 4-h incubation at 37°C, unbound cells were removed by washing, and the amount of adhered cells, solubilized by a 10 min treatment at room temperature (RT) with trypsin (Sigma T4799), was measured by flow cytometry (FC). FC measurements included a 4 h incubated inoculum (total cell) control, and controls with solvents and abiotic particles not incubated with cells (also to verify equal coating), also upon trypsin treatment.

For measuring adhesion to HeLa epithelial cells, yeast precultures were diluted in RPMI medium to a concentration of 6 × 10^3^ cells/ml. A total of 40 μl of each cell suspension were added to wells of a 12-well plate containing a confluent layer of HeLa cells, prepared as described previously (Fernández-Aroca et al. 2019), and incubated for 2 h at 37°C with 5% CO_2_. After this, unattached cells were collected and attached cells were scrapped off and collected in 2 ml of PBS for colony forming unit counting after plating on YPD (Reithofer et al. 2021). Each sample included a control without HeLa cells to correct for possible plastic adhesion.

### Virulence

Virulence of deletion mutants was tested using *Galleria mellonella* as *in vivo* invertebrate model of candidiasis, as described (Alvarado et al. 2024). Briefly, *G. mellonella* larvae, purchased from Artroposfera (Toledo, Spain), were selected and incubated for 24 h at 37°C to check their viability. Meanwhile, yeast pre-cultures were prepared, washed, and diluted in PBS to a concentration of 5 × 10^6^ cells/ml. A total of 10 µl of these suspensions were injected with a Hamilton syringe (Agilent) into the last left pseudopod of the larvae (15 larvae per group). Larvae injected with PBS or nonpathogenic *S. cerevisiae* (strain CEN.PK113-7D) were used as controls. Larvae were kept at 37°C, and their survival was monitored daily for one week. Data were collected from two independent blinded experiments, each with two biological replicates per yeast strain.

### Protein structure modeling

Three-dimensional structures of natural and mutated versions of Pir1 were modeled using the AlphaFold2 algorithm available at the ColabFold server (Mirdita et al. 2022), and subsequently visualized and aligned using RCSB Protein Data Bank webtools (https://www.rcsb.org/).

### Generation of polyclonal anti-Pir1 antiserum

Anti-Pir1 polyclonal antibodies were obtained against a recombinant Pir1 protein. The complete *PIR1* gene was amplified (for primers see Supplementary Table S1) and cloned in plasmid pET21b(+) (Novagen) for protein expression in *Escherichia coli* BL21 (DE3) (Thermo Fisher). The His-tagged protein was purified using HisTrap FF crude columns (GE Healthcare) following manufacturer´s instructions, and its purity was checked by SDS-PAGE. Antibodies against the purified protein were raised in adult female New Zealand White rabbits. After ammonium sulfate precipitation, IgGs in the serum were purified using Protein A HP SpinTrap columns (GE Healthcare).

### Pir1 immunoblot analysis

Cell walls, isolated as described (De Groot et al. 2004), were treated with ice-cold 30 mM NaOH o/n to extract alkali-labile CWPs. Solubilized proteins were neutralized with 30 mM acetic acid, separated on 3%–8% acrylamide Nu-PAGE gels (Thermo Fisher), and transferred to polyvinylidene difluoride membranes. After blocking (5% milk powder in PBS), blots were incubated with polyclonal anti-Pir1 antiserum. Following washing with PBS, membranes were incubated with secondary antibody (HRP-conjugated Goat anti-rabbit, Thermo Fisher), and developed using ECL Prime reagent (Amersham). Signals were captured using a luminescent image analyzer LAS 4000 (Fujifilm). Relative protein quantification was performed using ImageJ software (https://imagej.net/ij/).

### Pir1 cell wall localization by immunofluorescence staining

Overnight *C. albicans* cells were washed (PBS) and fixed in 4% paraformaldehyde for 30 min at RT. Washed cells (∼1 × 10^5^ cells) were incubated o/n under rotation at 4°C with polyclonal anti-Pir1 antiserum in PBS containing 3% BSA. Cells were then treated for 30 min at 4°C with Cy3-conjugated anti-rabbit IgG (Jackson ImmunoResearch) as secondary antiserum. In addition, cells were counterstained with CFW (10 µg/ml) for 5 min to label cell wall chitin. Fluorescently labeled cells were imaged using a Zeiss LSM 710 confocal laser scanning microscope, and data were analyzed with ZEN software (version 3.8.99.00000). Images were acquired at 8-bit depth and 1024 × 1024 resolution using a Plan-Apochromat 63×/1.40 Oil DIC M27 oil immersion objective.

### *PIR1* gene expression analysis by qPCR

*Candida albicans* cells were grown in YPD to exponential phase. Cells were disintegrated in the presence of TRIzol Reagent (Invitrogen) and 0.5 mm glass beads (Sartorius) using a Fast Prep-24 (MP Biomedicals) instrument. Purified RNA, isolated, quantified, and verified using standard methods, was treated with RNase-free DNase I (Thermo Fisher), and 2 µg were reverse-transcribed using a High-Capacity RNA-to-cDNA kit (Applied Biosystems). Twenty-fold diluted cDNA was used in qPCR reactions to analyze expression of *PIR1. PMA1* and *RPPB2* were applied as housekeeping genes for normalization. See Supplementary Table S1 for primers used in qPCR.

### Statistical analysis

Each assay was performed with at least two biological and two technical replicates unless otherwise stated. Statistical analysis was performed using GraphPad Prism 9 software (GraphPad Software) using one-way ANOVA for data sets with normal distribution followed by the post-hoc Tukey testing. Survival data was analyzed with the Kaplan-Meier method and compared with the Mantel–Cox Log-rank test. Differences with *P*-values < 0.05 were considered statistically significant.

## Results

### Pir1 mediates *in vitro* binding to β-1,3-glucan

Previous studies reported contradictory results concerning the relevance of the *C. albicans* proteins Pir1 and Pir32 for cell wall integrity and fitness (Martínez et al. 2004, Bahnan et al. 2012, Kim et al. 2022). To evaluate the importance of both proteins, homozygous single and double mutants, were generated in reference strain SC5314. The mutants for both genes were verified by PCR and immunoblotting (*pir1*Δ/Δ), see Fig. S1 for *pir1*Δ/Δ, as well as by WGS (*pir1*Δ/Δ) before phenotypic characterizations, mostly focusing on surface-related features, were executed.

**Figure 1.**
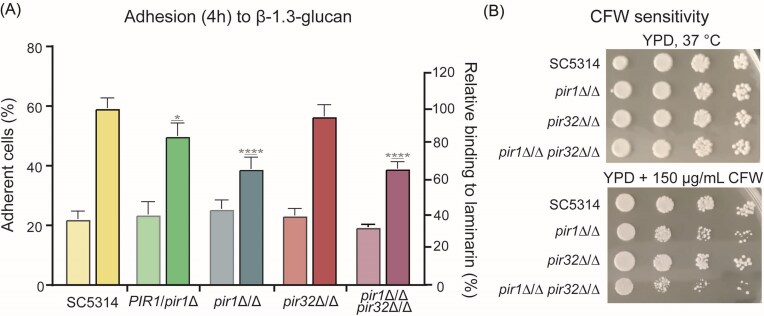
Phenotypic analysis of single and double *PIR1* and *PIR32* deletion mutants. (A) Adhesion to β-1,3-glucan (laminarin)-coated surface after 4 h of incubation (dark-colored bars). Adhesion to uncoated polystyrene surface was included as control (light-colored bars). Data are the mean ± SD of at least two biological replicates measured in triplicate. Asterisks indicate significant differences compared to laminarin-binding of SC5314 (*P < .05; ****, *P* < .0001). (B) Representative images of CFW sensitivity drop assay.

In line with its proposed binding to β-1,3-glucan, mutant cells lacking one or both *PIR1* alleles showed clearly reduced adhesion to laminarin. More precisely, the homozygous mutants, *pir1*Δ/Δ and *pir1*Δ/Δ *pir32*Δ/Δ, both showed a 34% reduction of laminarin binding compared to the parental strain while in the heterozygous mutant this was reduced by 16% (Fig. 1A). When conducting a battery of other phenotypic assays (Table 1), the homozygous mutants lacking *PIR1* (*pir1*Δ/Δ and *pir1*Δ/Δ *pir32*Δ/Δ) only showed a slightly increased sensitivity when they were grown in the presence of the wall perturbant CFW (Fig. 1B).

**Table 1.** Summary of phenotypic assays performed with *C. albicans PIR1* and *PIR32* deletion mutants.

Phenotypic assay	*pir1∆/∆*	*pir32∆/∆*	*pir1∆/∆ pir32∆/∆*
Growth rate	np^a^	np	np
Yeast morphology	np	np	np
Hyphae formation	np	np	np
Agar invasion	np	np	np
*In vivo* virulence (*G. mellonella*)	np	np	np
*Cell surface hydrophobicity*			
Logarithmic phase	96.5 ± 3.5^b^	96.2 ± 8.5	94.8 ± 3.0
Stationary phase	102.8 ± 7.9	103.2 ± 6.7	107.0 ± 0.7
*Sensitivity assays*			
Antifungals MIC	np	np	np
CR	np	np	np
Heat shock	np	np	np
NaCl	np	np	np
LiCl	np	np	np
Zymolyase	np	np	np
*Biofilm mass (CV assay)*			
YPD medium	101.4 ± 11.5	105.6 ± 10.5	104.7 ± 7.1
RPMI-1640	108.9 ± 7.0	102.8 ± 6.8	98.8 ± 7.9
*Adhesion*			
Polystyrene	115.1 ± 13.6	105.1 ± 10.7	87.9 ± 2.2
Pustulan	97.6 ± 10.7	100.9 ± 3.6	92.0 ± 6.1
Chitin	106.7 ± 1.3	104.0 ± 0.6	106.6 ± 2.6
HeLa cells	104.6 ± 8.1	98.8 ± 3.2	101.7 ± 7.7

a np, no phenotypic difference with SC5314.

b Numeric data relative to SC5314 normalized to 100%.

### The Pir1 structure: a core of antiparallel β-sheets surrounded by repeat units in (an) external loop(s)

**Figure 2.**
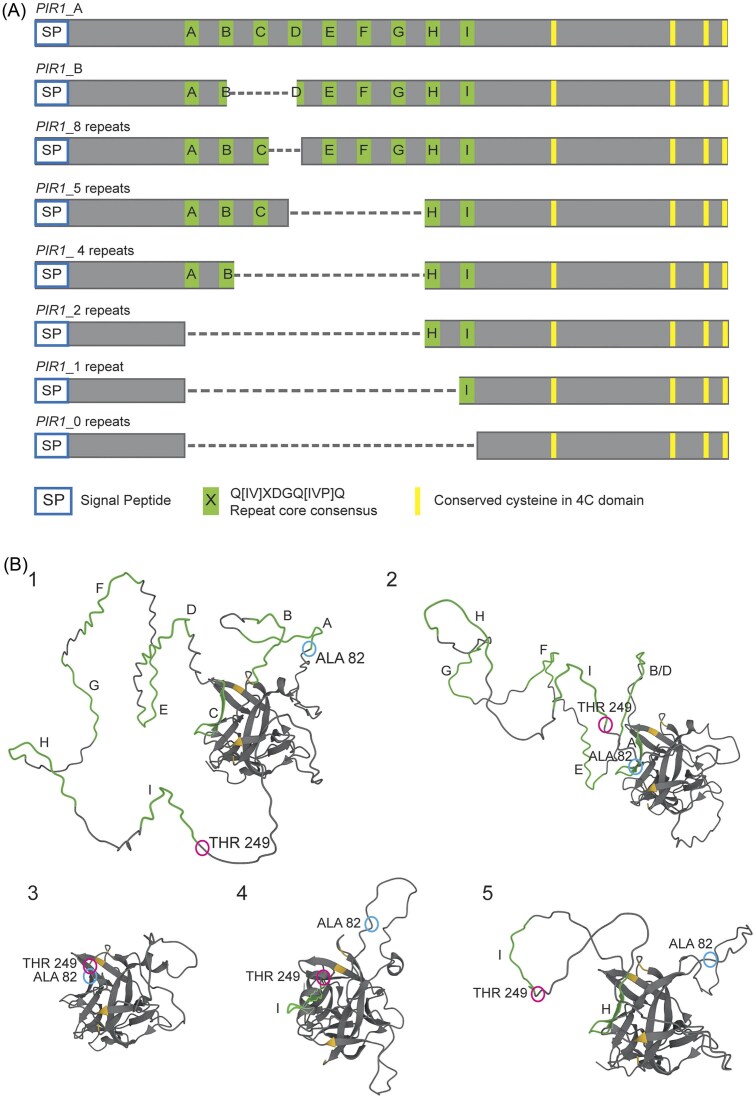
Modular organization (A) and structural comparison (B) of natural and synthetic *PIR1* genes. (A) Schematic alignment of *PIR1* alleles. *PIR 1*_A and *PIR1*_B correspond to alleles in SC5314, while *PIR1*_8 repeats, *PIR1*_5 repeats, and *PIR1*_4 repeats are from different clinical isolates (Fig. S2). Truncated constructs from *PIR1*_B with 0, 1, and 2 repeats are generated in this study. Note that a canonical Kex2 peptidase recognition site (KR) is absent in *C. albicans*. (B) Structural analysis (AlphaFold) of natural and truncated SC5314 alleles. 1, Pir1_A, 9 repeats; 2, Pir1_B, 7 repeats; 3–5, truncated Pir1 with 0, 1, and 2 repeats, respectively. Repeats units are highlighted in green, conserved cysteines in yellow. Ala82 and Thr249 bordering both sides of the repeat-containing sequence are indicated by circles. Numbers refer to corresponding amino acids in Pir1_A.

PCR analysis of about 250 clinical isolates obtained from local Spanish hospitals coincided with the observation by Kim et al. (2022), revealing *PIR1* alleles containing different numbers of repeats than the seven and nine observed in reference strain SC5314. Among our strain collections we detected isolates apparently containing between four and eleven repeats, however, most prevalent are strains containing at least one allele with nine repeats (91% in total), especially allelic combinations of 9/9 (39%), 9/8 (28%), and 9/7 (20%) repeats (Fig. S2). Sequence analysis of a few different allelic variants confirmed that the observed amplicon size differences are indeed due to variations in the number of internal repeats (Fig. S2 and Fig. 2A). Protein structure modeling of these natural alleles using AlphaFold revealed that Pir1 consists of a core domain composed of antiparallel β-sheets, which is stabilized by two disulfide bridges formed by the four conserved cysteines in the 4C domain. The core is surrounded by an unstructured loop where (most of) the repeat units are located (Fig. 2B). Curiously, all the protein models (Fig. 2B, see also Fig. S3) coincide in having one of the repeats located in between two β-sheets of the core domain instead of the external loop. With exception of the nine-repeat allele, it is the first repeat unit that is localized in the proximity of the protein core. In the nine-repeat allele, the third repeat unit (repeat C) is the one that is located between β-sheets of the protein core domain, consequently, repeats A and B form a separate small loop. Such a structural fold with (most of) the repeat units being accessible in (an) external loop(s) aligns perfectly with the idea that the repeat units in Pir proteins are responsible for the connection(s) to β-1,3-glucan(s).

To *in silico* analyze in more detail the relevance of the repeats in the Pir1 structure, truncated Pir1 proteins with 0, 1, and 2 repeat units were designed (Fig. 2A). As might be expected, in these truncated proteins, the core domain structure is largely unaffected, however, the outer loop where the repeats are localized shows large alterations. In the truncated protein without Pir repeats this loop is absent. Surprisingly, it is also absent in the truncated version containing only one repeat unit, which is placed between two β-sheets of the core domain. The truncated version with two repeats is the first containing a repeat unit in an outer loop (Fig. 2B). These data suggest that having a repeat unit as part of the core domain might be important for protein stability rather than or in addition to having a role in β-1,3-glucan binding.

### Two Pir1 repeats are necessary and sufficient for wall incorporation

**Figure 3.**
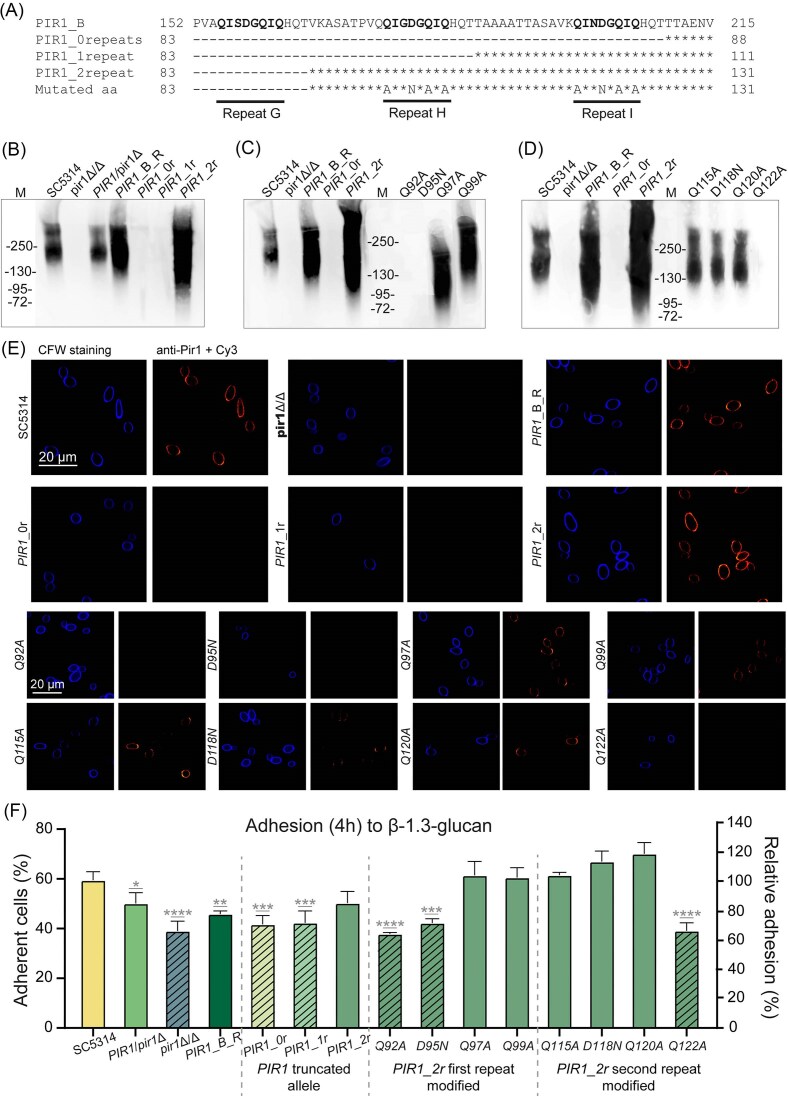
Two Pir repeat units are required and sufficient for covalent linkage of *C. albicans* Pir1 to the cell wall glucan network and phenotype recovery. (A) Schematic representation of the relevant part of mutant Pir1 proteins used in this study. (B) Immunoblot analysis of mild alkali-extracted wall proteins from *C. albicans* WT, homozygous, and heterozygous *pir1* deletion mutants, a *PIR1* short allele reintegrant strain, and reintegrants harboring truncated versions of Pir1 with 0,1 and 2 repeat units, respectively. (C and D) Analysis of point mutations in truncated Pir1 containing two repeats (Pir1_2r). Analysis of the first (C) and second (D) repeat are performed separately with control strains being added to both as reference on the left side of the blots. (E) Confocal immunofluorescence analysis of cell surface incorporation of Pir1. CFW co-staining was performed as control to mark cell wall chitin. (F) Laminarin-binding analysis. Data are the mean ± SD of at least two biological replicates measured in triplicate. Asterisks indicate significant differences to SC5314 (*P < .05; **P < .01; ***P < .001; ****P < .0001). Note the decreased adhesion to laminarin of strains that do not incorporate Pir1 in the cell wall (diagonal striped bars).

To test the importance of the repeat units for wall incorporation and confirm the observed *pir1* phenotypes, complementation mutants with different numbers of internal repeats were generated (S2 Table and Fig. 3A). First, reintegration of the complete *PIR1*_B allele (containing seven repeats) in the homozygous mutant restored Pir1 wall incorporation, showing the smeary pattern on Western blot (Fig. 3B) that is typical for this highly *O*-glycosylated protein (Kapteyn et al. 2000, De Groot et al. 2004), and Pir1 cell surface localization (Fig. 3E). It also reverted the Pir1-mediated laminarin binding to the level of the heterozygous mutant (Fig. 3F) and the CFW hypersensitivity (not shown).

Next, the generation of truncated versions of the *PIR1*_B allele allowed us to demonstrate the importance of the repeat units for Pir1 wall incorporation and functionality. Truncated Pir1 containing two repeat units was incorporated efficiently into the wall—reaching levels even higher than those in parental and reintegrant strains—and recovered the phenotypes of the null mutant (Fig. 3B, E, and F). However, protein versions without or with only one repeat unit were not covalently bound to the cell wall, were not detectable at the cell surface, and did not recover the laminarin-binding phenotype. Thus, two Pir1 repeats seem necessary and sufficient for wall incorporation and functionality. These data align well with the structural modeling data suggesting that two repeats are minimally required to yield an external loop with a repeat unit that enables intense contact with β-1,3-glucan.

To study the importance of specific amino acid residues in the repeats, site-directed mutagenesis was carried out with the two remaining repeats in the truncated Pir1_2r. Point mutations were generated at positions shown to be crucial for cell wall incorporation of Cis3/Pir4 in *S. cerevisiae* (Ecker et al. 2006). Mutated amino acids were the three glutamines as well as the aspartic acid residue in the conserved core of both repeats (Fig. 3A). Immunoblot analysis of alkali extracted wall proteins indicated that Pir1 wall incorporation was completely abolished by mutations Q92A and D95N in the first repeat and Q122A in the second repeat (Fig. 3C and D), demonstrating the importance of these residues. Q115A, D118N, and Q120A mutations in the second repeat yielded a low Pir1 level, and only Pir1_2r carrying Q97A and Q99A mutations were incorporated into the cell wall at levels comparable to parental strain SC5314. These immunoblotting data were corroborated by immunofluorescence imaging results showing surface localization of Q97A, Q99A, Q115A, D118N, and Q120A but absence of Q92A, D95N, and Q122A (Fig. 3E). The importance of the mutated amino acid positions for Pir1_2r incorporation and functionality was further supported by analysis of laminarin binding: where Pir1_2R carrying Q122A, Q92A, and D95N mutations behaved as the null mutant, laminarin binding of all other mutated proteins was indistinguishable from the parental strain (Fig. 3F).

Finally, to ascertain that the observed altered Pir1 wall incorporation was not due to gene expression changes introduced by genetic manipulation, *PIR1* expression levels were analyzed by qPCR, showing that *PIR1* transcript levels of all site-directed mutants was comparable to the unmutated Pir1_2r strain and the heterozygous mutant (Fig. S4). Altogether, we conclude that the presence of at least two repeat units is essential for covalent Pir1 wall incorporation, and our study pinpoints the special relevance of residues Q92 and D95 in the first repeat and Q122 in the second repeat of our truncated Pir1-2r protein.

## Discussion

In this study, we have analyzed the functionality and wall incorporation of Pir1, a covalently bound alkali-labile CWP in *C. albicans* that is proposed to fulfill an important role in cell integrity by strengthening the polysaccharide matrix of the wall. Homozygous deletion of the *PIR1* gene in *C. albicans* neither affected the growth rate and morphology nor led to other severe cell surface-related phenotypes or changes in antifungal drug sensitivities, consistent with the study by Kim *et al*. (2022). In assays with cell wall perturbing agents, only when adding CFW a slight hypersensitivity of mutants lacking both alleles of the *PIR1* gene was observed. Additional deletion of *PIR32* did not aggravate phenotypes observed for *PIR1* deletion mutants, which is also consistent with Kim *et al*. (2022). Our data contrast with an older study in which heterozygous *PIR1* mutants showed strong morphological and drug-sensitivity phenotypes (Martínez et al. 2004), possibly owing to undesired genomic alterations introduced when using the *URA*-blaster disruption system (Brand et al. 2004, Correia et al. 2010).

Because of its proposed role as cross-linker of β-1,3-glucan molecules, we also tested *in vitro* binding of wild type and mutant cells to immobilized β-1,3-glucan (laminarin) and other cell wall molecules (β-1,6-glucan and chitin). In line with its proposed function, mutants lacking Pir1 indeed showed significantly diminished binding to β-1,3-glucan, whereas binding to β-1,6-glucan or chitin was unaltered. Reintegration of the short *PIR1* allele (seven repeat units) in the *pir1* null mutant recovered the presence of the protein in the cell wall as well as the laminarin-binding phenotype of the mutant.

Pir proteins are historically described as secretory proteins that contain at least one Q[IV]XDGQ[IVP]Q repeat unit as well as a conserved C-terminal 4C domain (De Groot et al. 2005). *Candida albicans* contains two such protein-encoding genes, *PIR1* and *PIR32*. Previous studies with *PIR* gene deletants in *S. cerevisiae* have shown their importance for cellular growth, morphology, and mating, especially when simultaneously deleting multiple *PIR* genes (Mrsă and Tanner 1999). As such, lack of strong phenotypes for *C. albicans pir1 pir32* double mutants led Kim *et al*. to suggest that the Pir family in *C. albicans* might be expanded with secretory proteins minimally containing a DGQ motif together with a downstream QFQFD motif (Kim et al. 2022). However, no experimental evidence for this has been provided to date and the newly suggested proteins have never been identified in purified cell wall extracts. Nonetheless, absence of cell wall weakening of homozygous *pir1 pir32* double deletion mutants is very surprising if the protein is important for wall strength. Even more so because *Saccharomycetaceae* such as *S. cerevisiae* and *C. glabrata*, but not *C. albicans*, contain a few GPI proteins with single Pir repeat units that are linked to both β-1,6-glucan and β-1,3-glucan (Kapteyn et al. 2001) and thus may provide further cell wall stability.

Pir32 contains only one Pir repeat unit. Consistent with the absence of any cell surface-related phenotypes in its deletion mutant, the protein has never been detected in wall proteomic studies. Moreover, gene expression studies did not show any upregulation of *PIR32* to compensate for the loss of Pir1 (data not shown). Our further investigations therefore focused on the wall incorporation and functionality of *C. albicans* Pir1, a classical mild alkali-sensitive CWP with 4–11 repeat units observed in clinical isolates that is consistently being detected in cell wall preparations (De Groot et al. 2004, Sosinska et al. 2011, Sorgo et al. 2013). Our mutagenesis approach to study Pir1 wall incorporation was based on an elegant study by Ecker and colleagues (Ecker et al. 2006) focusing on *S. cerevisiae* Cis3/Pir4, a small Pir protein containing two repeats although the second was described as “pseudo” because it is somewhat atypical (QATDSQAQ) and was shown not to be essential for Pir4 wall incorporation. Applying Edman degradation sequencing of wall-incorporated protein, they demonstrated the presence of an alkali-labile ester bond between a glutamic acid residue in the first repeat and β-1,3-glucan (Ecker et al. 2006). As Pir1 in *C. albicans* lacks the typical Kex2 protease recognition site KR and in a cell wall proteomic study has been shown to remain (at least in part) unprocessed (De Groot et al. 2004), Edman degradation will not reveal the link between the first repeat unit and β-1,3-glucan.

Because truncated *S. cerevisiae* Pir4 comprising only one repeat unit gets covalently incorporated in the wall, we tested whether truncated *C. albicans* Pir1 versions containing zero, one or two repeats could be wall incorporated and would recover laminarin binding. The two-repeats version of Pir1 (Pir1_2r) was efficiently incorporated in the wall as demonstrated by its presence in alkali extracts and surface immunolabeling reaching levels that were even higher than in the wild-type strain. Proper functionality of this protein was indicated by the restoration of laminarin binding to levels comparable to strains containing one functional *PIR1* allele (the heterozygous mutant and the reintegrant strain). Thus, where in the case of *Sc*Pir4, one repeat unit is sufficient for covalent protein incorporation, our data indicate that *C. albicans* Pir1 requires two repeats for its covalent connection to the cell wall. Protein modeling suggests that one of the repeat units always forms part of the protein core, and at least two copies of the repeat are needed to form an external loop with (a) repeat(s) that might provide better access to β-1,3-glucan.

**Figure 4.**
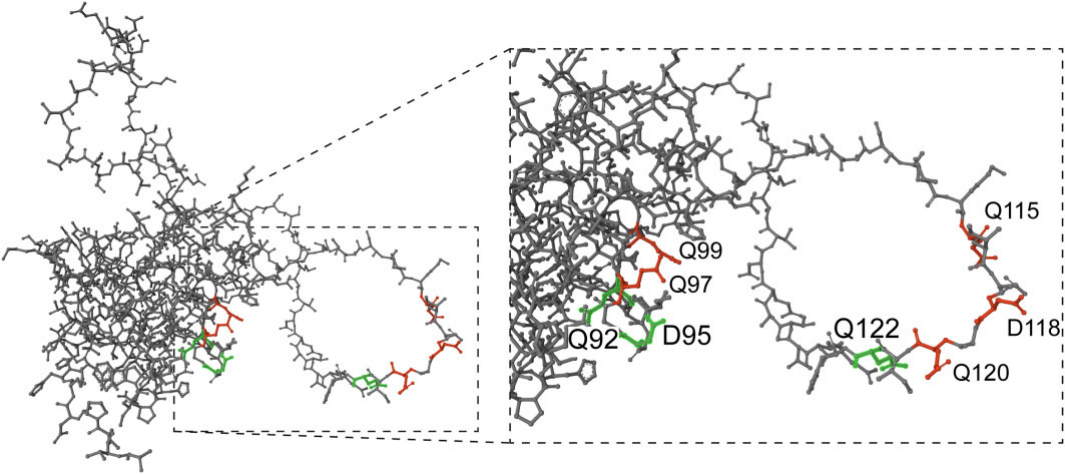
Ball-and-stick model of the synthetic Pir1_2r protein. Indicated with green and red colors are conserved repeat residues that are mutated in this study. In green, residues that are essential for Pir1_2r wall incorporation.

Using site-directed mutagenesis, Ecker *et al*. also showed that four amino acid residues in the (first) repeat of *S. cerevisiae* Pir4, the three glutamines and the aspartic acid, were crucial for covalent wall incorporation (Ecker et al. 2006). Guided by this, we substituted these amino acids in both repeat units of the Pir1_2r protein. Results obtained with these mutants were generally consistent with the study of Ecker *et al*. with some minor differences. Like in their paper, mutation of the first glutamine (Q92) and aspartic acid (D95) in the first repeat totally abolished wall incorporation. However, mutated proteins Q97A and Q99A were incorporated in the wall, albeit not to the level of the Pir-2r protein. In the second repeat, the last Q122A mutation totally impeded wall incorporation, whereas Q115A, D118N, and Q120A mutations resulted in low levels of wall-incorporated Pir1. Absence of Pir1 in walls of Q92A, D95N, and Q122A mutants coincided with lowered laminarin binding as observed in the null mutant, whereas all other mutants were indistinguishable from the parental strain, also suggesting that a high level of Pir1 in the cell wall is not required to achieve a basal level of Pir1 functionality. In conclusion, in contrast to *Sc*Pir4, *C. albicans* Pir1 incorporation seems to have a minimal requirement of a two-repeat module in which especially the first glutamine and aspartate in the first repeat and the last glutamine in the second repeat are crucial for protein incorporation and functionality (Fig. 4). As these three residues are relatively in close proximity to each other, we could speculate that perhaps they allow interaction of β-1,3-glucan with both repeats, however, this needs further investigation.

AlphaFold structural predictions tend to be reliable, but we are aware that one must be careful in drawing conclusions, especially when considering regions that lack secondary structures such as the Pir1 loop(s). Moreover, the Pir1 structure is likely to be influenced by its heavy glycosylation, which increases its mass in a heterogenous manner up to about 300 kDa, as visualized on immunoblots. The same high mass was also observed previously when probing wall extracts with cross-reactive anti-*Sc*Pir2 antiserum, and has been described to be due to *N*- as well as *O*-glycan additions (Kapteyn et al. 2000). Remarkably, removing five repeat units did not lower the Mr of the glycosylated protein smear on blots suggesting that most of the glycan is added to residues that are untouched by this truncation. Conversely, the Q97A mutation in the first repeat of Pir1_2r lowered its Mr by about 50 kDa. At this point, we can only speculate that this mutation perhaps alters the protein fold in a way that part of the protein may become less accessible to protein glycosyl transferase enzymes during secretion without affecting β-1,3-glucan accessibility and binding.

Looking further into Pir repeat unit sequences, we noticed that in comparison to Pir32 and the five *S. cerevisiae* Pir proteins all *C. albicans* Pir1 repeats have a HQT extension and follow the consensus QIXDGQ[IV]QHQT. Whether this addition, especially the extra glutamine, plays a role in Pir1 incorporation remains to be studied.

In conclusion, our study provides mechanistic information on how *C. albicans* Pir1 is covalently linked to cell wall β-1,3-glucan to foster cellular stability, and demonstrates the key importance of the Pir repeat units for wall incorporation. Although it is remarkable that 91% of the isolates analyzed from our collections harbor at least one nine-repeat allele of *PIR1*, the number of repeat units may vary between isolates. In view of the importance of Pir proteins for cellular integrity in *S. cerevisiae* (Mrsă and Tanner 1999), especially in case of cell wall stress (Kapteyn et al. 2001), and the absence of GPI proteins with Pir repeat units in *C. albicans*, it is surprising that *pir1* null mutants hardly showed growth defects. We hypothesize that other types of CWPs, for instance, transglycosidases of Bgl2, Gas/Phr, Crh families or others may have taken on a more prominent role in cross-linking cell wall polysaccharides of *C. albicans* to accomplish the needed cell wall strength.

## Supplementary Material

foaf042_Supplemental_Files

## References

[bib1] Alvarado M, Gómez-Navajas JA, Blázquez-Muñoz MT et al. The good, the bad, and the hazardous: comparative genomic analysis unveils cell wall features in the pathogen *Candidozyma auris* typical for both baker's yeast and *Candida*. FEMS Yeast Res. 2024;24:foae039. 10.1093/femsyr/foae039. 39656857 PMC11657238

[bib2] Bahnan W, Koussa J, Younes S et al. Deletion of the *Candida albicans PIR32* results in increased virulence, stress response, and upregulation of cell wall chitin deposition. Mycopathologia. 2012;174:107–19. 10.1007/s11046-012-9533-z. 22391823

[bib3] Brand A, MacCallum DM, Brown AJP et al. Ectopic expression of *URA3* can influence the virulence phenotypes and proteome of *Candida albicans* but can be overcome by targeted reintegration of *URA3* at the *RPS10* locus. Euk Cell. 2004;3:900–9. 10.1128/EC.3.4.900-909.2004. PMC500875 15302823

[bib4] Butler G, Rasmussen MD, Lin MF et al. Evolution of pathogenicity and sexual reproduction in eight *Candida* genomes. Nature. 2009;459:657–62. 10.1038/nature08064. 19465905 PMC2834264

[bib5] Correia A, Lermann U, Teixeira L et al. Limited role of secreted aspartyl proteinases Sap1 to Sap6 in *Candida albicans* virulence and host immune response in murine hematogenously disseminated candidiasis. Infect Immun. 2010;78:4839–49. 10.1128/IAI.00248-10. 20679440 PMC2976357

[bib6] Dadar M, Tiwari R, Karthik K et al. *Candida albicans* —Biology, molecular characterization, pathogenicity, and advances in diagnosis and control—An update. Microb Pathog. 2018;117:128–38. 10.1016/j.micpath.2018.02.028. 29454824

[bib7] De Groot PWJ, De Boer AD, Cunningham J et al. Proteomic analysis of *Candida albicans* cell walls reveals covalently bound carbohydrate-active enzymes and adhesins. Euk Cell. 2004;3:955–65. 10.1128/EC.3.4.955-965.2004. PMC500891 15302828

[bib8] De Groot PWJ, Ram AF, Klis FM. Features and functions of covalently linked proteins in fungal cell walls. Fungal Genet Biol. 2005;42:657–75. 10.1016/j.fgb.2005.04.002. 15896991

[bib9] Ecker M, Deutzmann R, Lehle L et al. Pir proteins of *Saccharomyces cerevisiae* are attached to beta-1,3-glucan by a new protein-carbohydrate linkage. J Biol Chem. 2006;281:11523–9. 10.1074/jbc.M600314200. 16495216

[bib10] Ene IV, Heilmann CJ, Sorgo AG et al. Carbon source-induced reprogramming of the cell wall proteome and secretome modulates the adherence and drug resistance of the fungal pathogen *Candida albicans*. Proteomics. 2012;12:3164–79. 10.1002/pmic.201200228. 22997008 PMC3569869

[bib11] EUCAST . EUCAST antifungal MIC method for yeasts. 2023. https://www.eucast.org/fileadmin/src/media/PDFs/EUCAST_files/AFST/Files/EUCAST_E.Def_7.4_Yeast_definitive_revised_2023.pdf.

[bib12] Fernández-Aroca DM, Roche O, Sabater S et al. P53 pathway is a major determinant in the radiosensitizing effect of Palbociclib: implication in cancer therapy. Cancer Lett. 2019;451:23–33. 10.1016/j.canlet.2019.02.049. 30872077

[bib13] Fernández-Pereira J, Alvarado M, Gómez-Molero E et al. Characterization of Awp14, a novel cluster III adhesin identified in a high biofilm-forming *Candida glabrata* isolate. Front Cell Infect Microbiol. 2021;11:790465. 10.3389/fcimb.2021.790465. 34869084 PMC8634165

[bib14] Grahl N, Demers EG, Crocker AW et al. Use of RNA-protein complexes for genome editing in Nnn-*albicans Candida* species. mSphere. 2017;2:e00218–00217. 10.1128/mSphere.00218-17. 28657070 PMC5480035

[bib15] Grbavac A, Canak I, Stuparevic I et al. Proteolytic processing of the *Sac* *charomyces cerevisiae* cell wall protein Scw4 regulates its activity and influences its covalent binding to glucan. Biochim Biophys Acta Mol Cell Res. 2017;1864:507–15. 10.1016/j.bbamcr.2016.12.009. 27965112

[bib16] Heilmann CJ, Sorgo AG, Mohammadi S et al. Surface stress induces a conserved cell wall stress response in the pathogenic fungus *Candida albicans*. Euk Cell. 2013;12:254–64. 10.1128/EC.00278-12. PMC3571293 23243062

[bib17] Kapteyn JC, Hoyer LL, Hecht JE et al. The cell wall architecture of *Candida albicans* wild-type cells and cell wall-defective mutants. Mol Microbiol. 2000;35:601–11. 10.1046/j.1365-2958.2000.01729.x. 10672182

[bib18] Kapteyn JC, Ter Riet B, Vink E et al. Low external pH induces *HOG1*-dependent changes in the organization of the *Saccharomyces cerevisiae* cell wall. Mol Microbiol. 2001;39:469–80. 10.1046/j.1365-2958.2001.02242.x. 11136466

[bib19] Kim J, Oh SH, Rodriguez-Bobadilla R et al. Peering into *Candida albicans* Pir protein function and comparative genomics of the Pir family. Front Cell Infect Microbiol. 2022;12:836632. 10.3389/fcimb.2022.836632. 35372132 PMC8975586

[bib20] Klis FM, Boorsma A, De Groot PWJ. Cell wall construction in *Saccharomyces cerevisiae*. Yeast. 2006;23:185–202. 10.1002/yea.1349. 16498706

[bib21] Klis FM, de Groot P, Hellingwerf K. Molecular organization of the cell wall of *Candida albicans*. Med Mycol. 2001;39:1–8. 10.1080/mmy.39.1.1.8-0. 11800263

[bib22] Klis FM, Sosinska GJ, de Groot PWJ et al. Covalently linked cell wall proteins of *Candida albicans* and their role in fitness and virulence. FEMS Yeast Res. 2009;9:1013–28. 10.1111/j.1567-1364.2009.00541.x. 19624749

[bib23] Martínez AI, Castillo L, Garcera A et al. Role of Pir1 in the construction of the *Candida albicans* cell wall. Microbiology. 2004;150:3151–61. 10.1099/mic.0.27220-0. 15470096

[bib24] Masuoka J. Surface glycans of *Candida albicans* and other pathogenic fungi: physiological roles, clinical uses, and experimental challenges. Clin Microbiol Rev. 2004;17:281–310. 10.1128/CMR.17.2.281-310.2004. 15084502 PMC387410

[bib25] Miramon P, Lorenz MC. A feast for *Candida*: metabolic plasticity confers an edge for virulence. PLoS Pathog. 2017;13:e1006144. 10.1371/journal.ppat.1006144. 28182769 PMC5300112

[bib26] Mirdita M, Schutze K, Moriwaki Y et al. ColabFold: making protein folding accessible to all. Nat Methods. 2022;19:679–82. 10.1038/s41592-022-01488-1. 35637307 PMC9184281

[bib27] Mixao V, Gabaldon T. Genomic evidence for a hybrid origin of the yeast opportunistic pathogen *Candida albicans*. BMC Biol. 2020;18:48. 10.1186/s12915-020-00776-6. 32375762 PMC7204223

[bib28] Moreno-Ruiz E, Ortu G, De Groot PWJ et al. The GPI-modified proteins Pga59 and Pga62 of *Candida albicans* are required for cell wall integrity. Microbiology (Reading). 2009;155:2004–20. 10.1099/mic.0.028902-0. 19383685

[bib29] Mormeneo M, Andres I, Bofill C et al. Efficient secretion of *Bacillus subtilis* lipase A in *Saccharomyces cerevisiae* by translational fusion to the Pir4 cell wall protein. Appl Microbiol Biotechnol. 2008;80:437–45. 10.1007/s00253-008-1549-4. 18626643

[bib30] Mrsă V, Seidl T, Gentzsch M et al. Specific labelling of cell wall proteins by biotinylation. Identification of four covalently linked *O*-mannosylated proteins of *Saccharomyces cerevisiae*. Yeast. 1997;13:1145–54. 10.1002/(SICI)1097-0061(19970930)13:12<1145::AID-YEA163>3.0.CO;2-Y. 9301021

[bib31] Mrsă V, Tanner W. Role of NaOH-extractable cell wall proteins Ccw5p, Ccw6p, Ccw7p and Ccw8p (members of the Pir protein family) in stability of the *Saccharomyces cerevisiae* cell wall. Yeast. 1999;15:813–20. 10.1002/(SICI)1097-0061(199907)15:10A<813::AID-YEA421>3.0.CO;2-Y. 10407261

[bib32] Murad AM, Lee PR, Broadbent ID et al. CIp10, an efficient and convenient integrating vector for *Candida albicans*. Yeast. 2000;16:325–7. 10.1002/1097-0061(20000315)16:4<325::AID-YEA538>3.0.CO;2-. 10669870

[bib33] Odds FC, Brown AJ, Gow NA. *Candida albicans* genome sequence: a platform for genomics in the absence of genetics. Genome Biol. 2004;5:230. 10.1186/gb-2004-5-7-230. 15239821 PMC463275

[bib34] Reithofer V, Fernández-Pereira J, Alvarado M et al. A novel class of *Candida glabrata* cell wall proteins with β-helix fold mediates adhesion in clinical isolates. PLoS Pathog. 2021;17:e1009980. 10.1371/journal.ppat.1009980. 34962966 PMC8746771

[bib35] Reuß O, Vik A, Kolter R et al. The *SAT1* flipper, an optimized tool for gene disruption in *Candida albicans*. Gene. 2004;341:119–27. 10.1016/j.gene.2004.06.021. 15474295

[bib36] Sardi JCO, Scorzoni L, Bernardi T et al. *Candida* species: current epidemiology, pathogenicity, biofilm formation, natural antifungal products and new therapeutic options. J Med Microbiol. 2013;62:10–24. 10.1099/jmm.0.045054-0. 23180477

[bib37] Schille TB, Sprague JL, Naglik JR et al. Commensalism and pathogenesis of *Candida albicans* at the mucosal interface. Nat Rev Micro. 2025;23:525–40. 10.1038/s41579-025-01174-x. 40247134

[bib38] Smits GJ, Kapteyn JC, Van den Ende H et al. Cell wall dynamics in yeast. Curr Opin Microbiol. 1999;2:348–52. 10.1016/S1369-5274(99)80061-7. 10458981

[bib39] Sorgo AG, Brul S, De Koster CG et al. Iron restriction-induced adaptations in the wall proteome of *Candida albicans*. Microbiology (Reading). 2013;159:1673–82. 10.1099/mic.0.065599-0. 23728625

[bib40] Sorgo AG, Heilmann CJ, Dekker HL et al. Effects of fluconazole on the secretome, the wall proteome, and wall integrity of the clinical fungus *Candida albicans*. Euk Cell. 2011;10:1071–81. 10.1128/EC.05011-11. PMC3165447 21622905

[bib41] Sosinska GJ, De Koning LJ, De Groot PWJ et al. Mass spectrometric quantification of the adaptations in the wall proteome of *Candida albicans* in response to ambient pH. Microbiology (Reading). 2011;157:136–46. 10.1099/mic.0.044206-0. 20864472

[bib42] World Health Organization . WHO Fungal Priority Pathogens List to Guide Research, Development and Public Health Action. 2022. www.who.int/publications/i/item/9789240060241.

[bib43] Zakrzewska A, Boorsma A, Brul S et al. Transcriptional response of *Saccharomyces cerevisiae* to the plasma membrane-perturbing compound chitosan. Euk Cell. 2005;4:703–15. 10.1128/EC.4.4.703-715.2005. PMC1087819 15821130

