# Supplementary figures and images for: Wall incorporation of the β-1,3-glucan cross-linking protein Pir1 in the human pathogen Candida albicans is facilitated by the presence of two or more Pir repeat units
Source: FEMS Yeast Res. 2025 Aug 7;25:foaf042. doi: 10.1093/femsyr/foaf042 (PMC12359137; doi:10.1093/femsyr/foaf042)

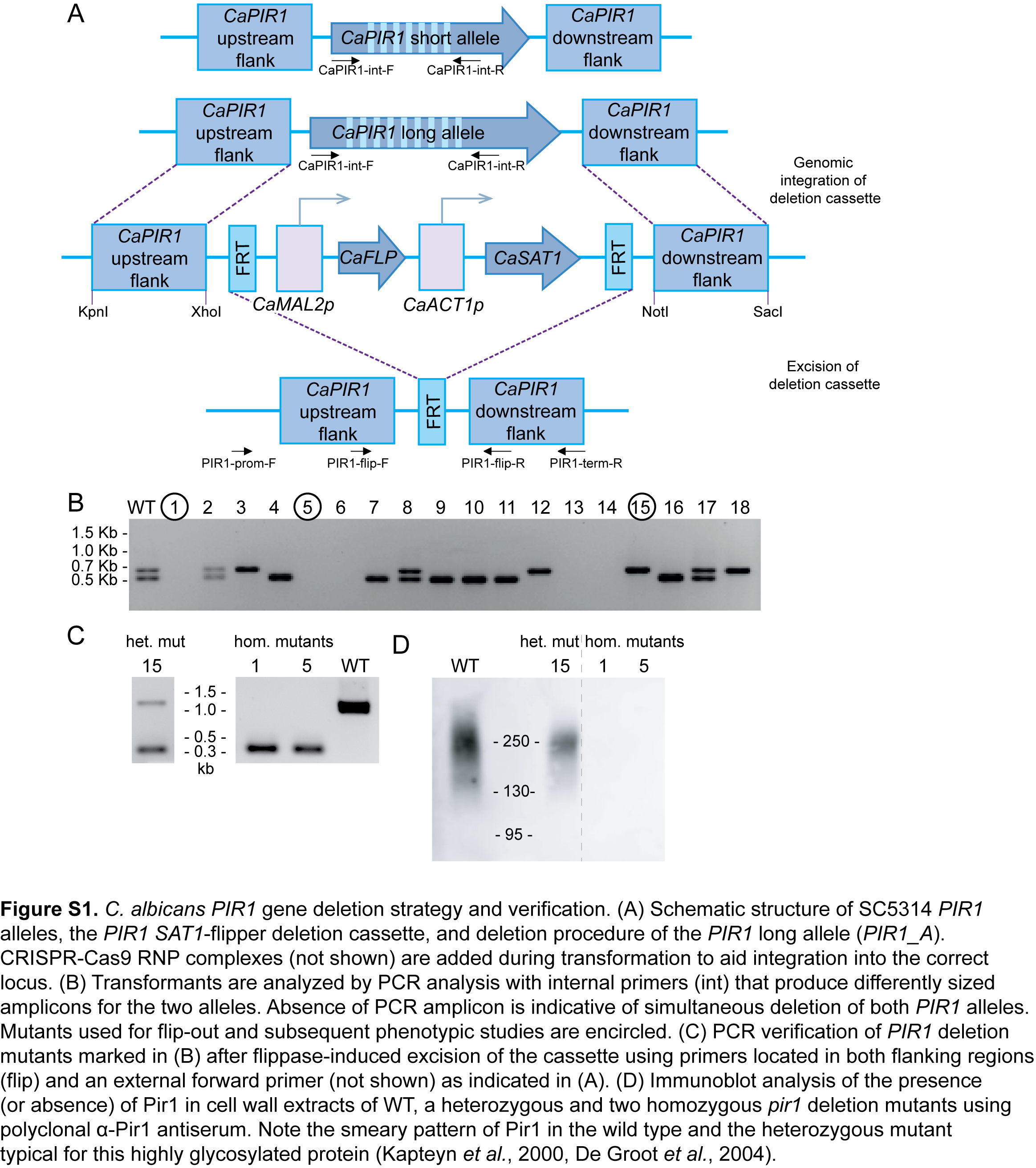

Supplement: foaf042_Supplemental_Files [file foaf042_supplemental_files.zip › Figure S1.tif]

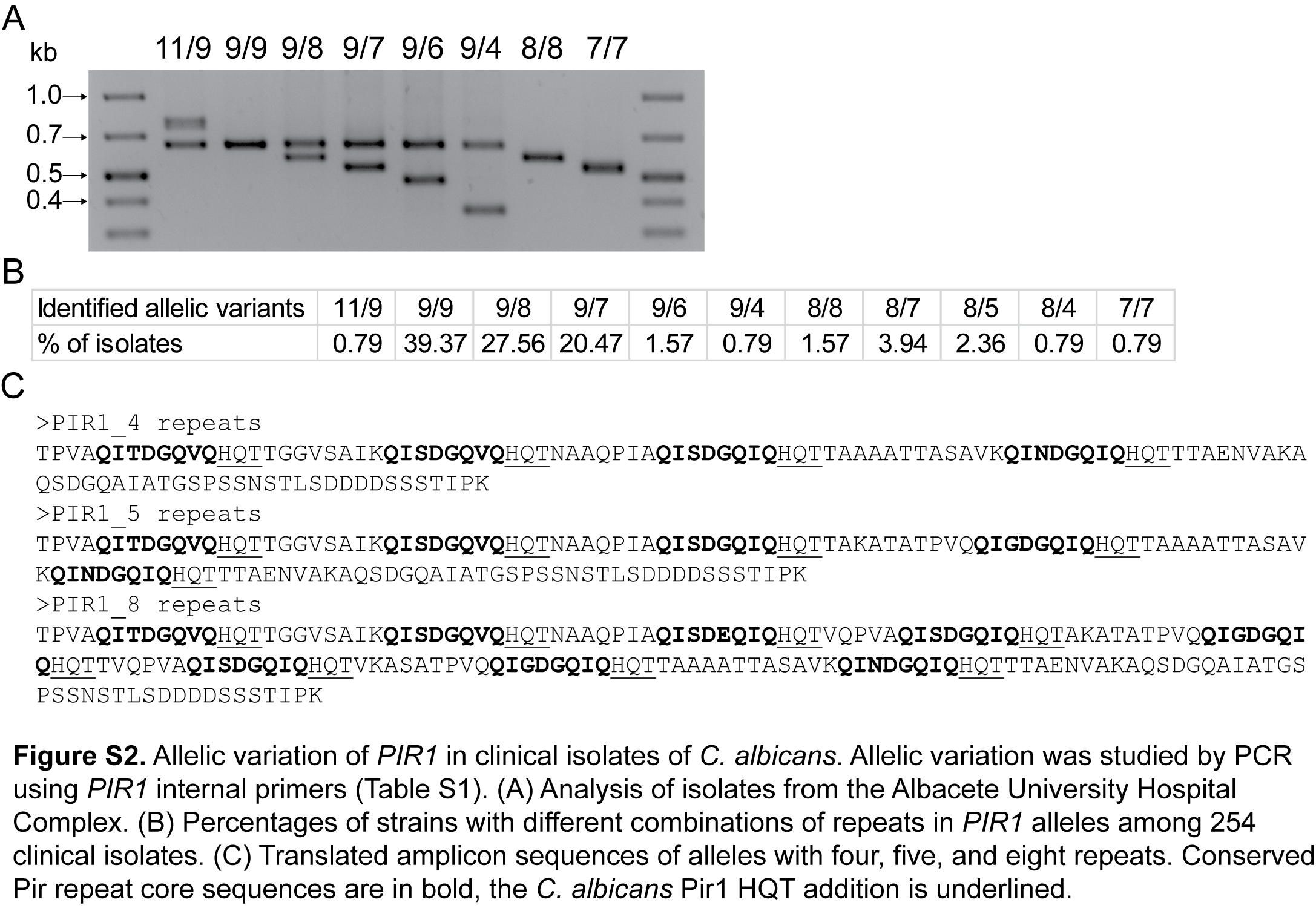

Supplement: foaf042_Supplemental_Files [file foaf042_supplemental_files.zip › Figure S2.tif]

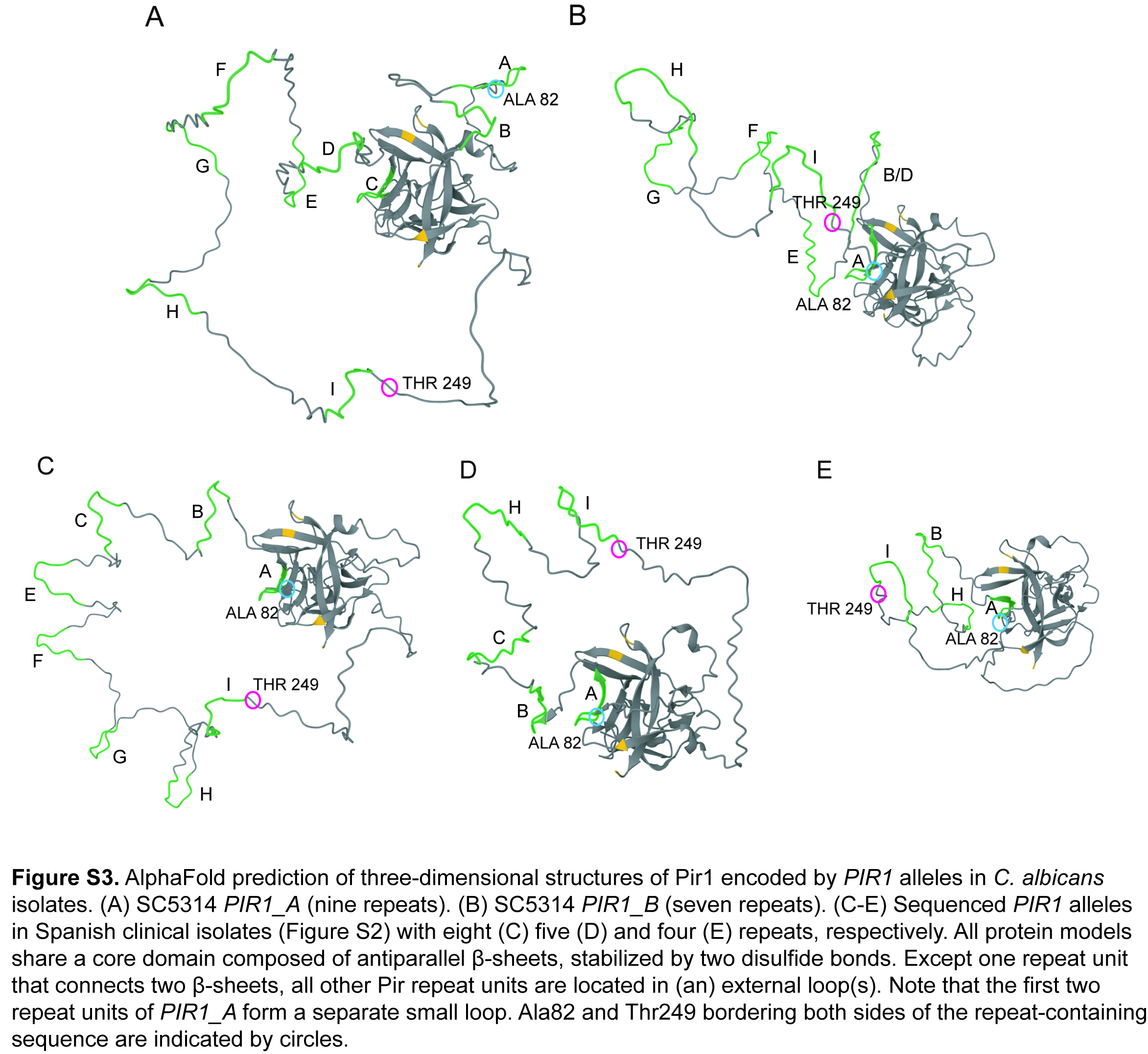

Supplement: foaf042_Supplemental_Files [file foaf042_supplemental_files.zip › Figure S3.tif]

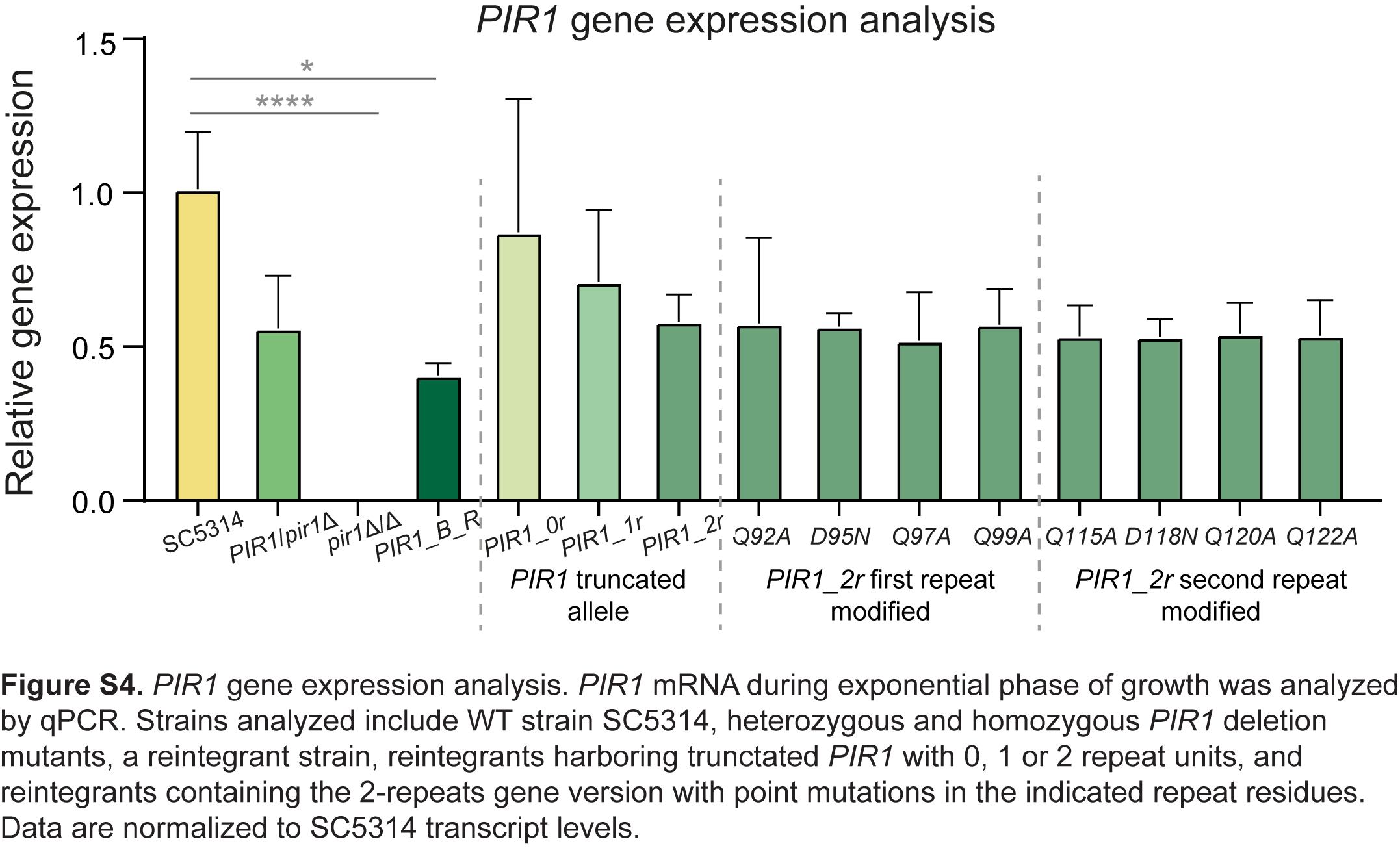

Supplement: foaf042_Supplemental_Files [file foaf042_supplemental_files.zip › Figure S4.tif]
